# Sustained efficacy of spesolimab following flare-up dose in achieving long-term skin clearance in generalized pustular psoriasis: A case report and review of literature

**DOI:** 10.1016/j.jdcr.2025.10.059

**Published:** 2025-11-07

**Authors:** Abdullah Faisal Albadri, Abdullah Ayman Aman, Abdulrahman Esam Azhar, Saif Yaseer Ashram

**Affiliations:** a Department of Dermatology, Consultant and Head of Dermatology at King Fahd General Hospital, Jeddah, Saudi Arabia; b Dermatology Service Lead at Ministry of Health, Jeddah, Saudi Arabia; c Department of Dermatology, German Board & Fellowship in Allergy, Vivantes Hospital, Charite University, Berlin, Germany; d Department of Dermatology, Specialist Dermatology MD at King Fahd General Hospital, Jeddah, Saudi Arabia; e Department of Dermatology, General Physician MD at King Fahd General Hospital, Jeddah, Saudi Arabia; f Department of Dermatology, Dermatology Resident MD at King Fahd General Hospital, Jeddah, Saudi Arabia

**Keywords:** acute flare, biologics, dermatopathology, generalized pustular psoriasis, GPP, IL-36, long term efficacy, spesolimab

## Introduction

Generalized pustular psoriasis (GPP) is a rare and potentially life-threatening form of psoriasis characterized by the sudden onset of sterile pustules on erythematous skin, often accompanied by systemic symptoms such as fever, malaise, and fatigue.^1^^,^^2^ Unlike plaque psoriasis, GPP is driven primarily by dysfunction of the interleukin-36 (IL-36) pathway.^3^ Traditional systemic therapies have often provided limited efficacy, underscoring the need for more targeted treatment strategies.^1^

Spesolimab, an IL-36 receptor inhibitor, has recently emerged as a promising therapy, demonstrating rapid resolution of GPP flares.^4^ We present the case of a 59-year-old woman with long-standing psoriatic arthritis (PsA) and GPP who experienced marked improvement and sustained remission following a single intravenous infusion of spesolimab.

## Case presentation

A 59-year-old female, known to have psoriatic arthritis (PsA) for the past 20 years and type 2 diabetes mellitus for 1 year, presented as a follow-up case of GPP. She was seen in the dermatology clinic on the 8th and 29th of December 2024, corresponding to 10- and 31-days following administration of a flare-up dose of Spesolimab (900 mg IV). The patient has a history of recurrent, episodic, annular, and pruritic skin lesions that began in 2019, which were often associated with vesicle formation. The eruptions were distributed across the entire body, primarily involving the abdomen, back, and thighs, with sparing of the face, palms, and soles. The skin lesions were temporarily alleviated by topical treatments, including mometasone and Fucidin creams. No specific aggravating factors were identified, and she reported multiple similar episodes over the years.

Her past medical history was notable for PsA and diabetes, for which she was on oral hypoglycemic therapy (metformin 500 mg once daily). She had no known allergies and no history of prior hospitalizations. Family history was unremarkable for dermatological conditions. Socially, she was a non-smoker, of low socioeconomic background, with no recent travel history. Her Dermatology Life Quality Index (DLQI) score was 21, reflecting a significant impact on her quality of life.

**Fig 1:**
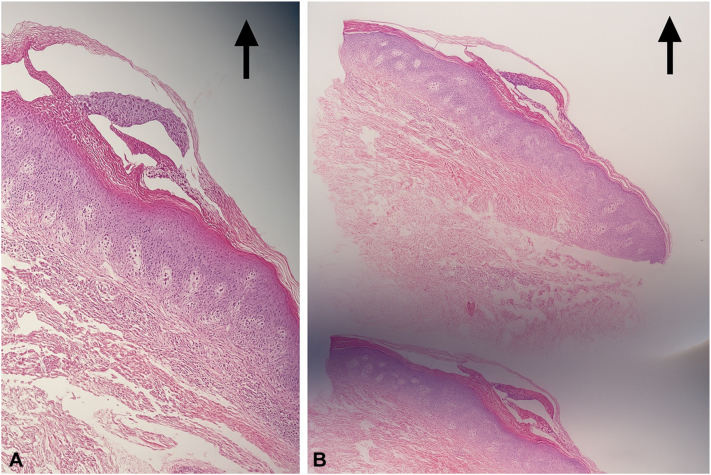
**A,** (hematoxylin and eosin, 10×) and **(B)** (hematoxylin and eosin, 4×). Histopathology showing regular acanthosis with a prominent sub-corneal neutrophilic collection and patchy parakeratosis.

Laboratory investigations at baseline were unremarkable, with normal complete blood count, liver function tests, renal function tests, and negative serology. C-reactive protein was elevated at 17 mg/L, indicating systemic inflammation. Screening for tuberculosis, including purified protein derivative and chest X-ray, was negative. A prior skin biopsy taken from the right side of the abdomen during an earlier follow-up visit confirmed the diagnosis of GPP (Fig 1, *A* and *B*).

**Fig 2:**
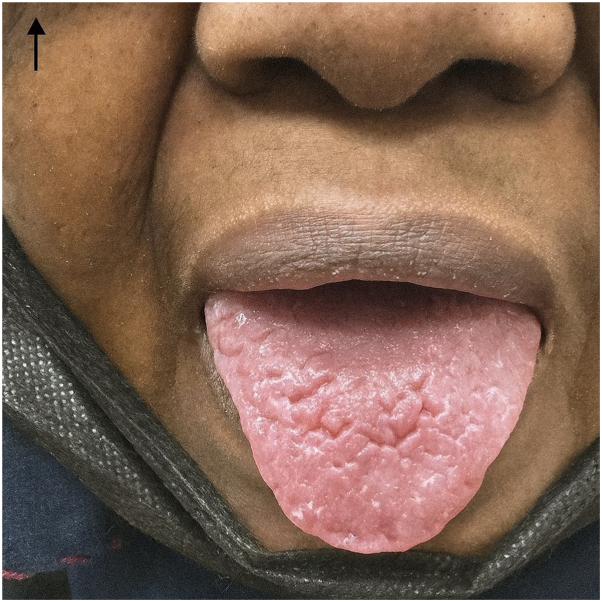
Fissured tongue as presented on oral cavity physical exam.

She reported systemic symptoms of fatigue and malaise, though there were no signs of fever, headache, or anorexia. On mucosal examination, a fissured tongue was noted (Fig 2), while there was no ocular or gastrointestinal involvement, and the nails appeared normal.

Prior to the administration of Spesolimab, on the 28th of November, physical examination revealed the presence of sterile, non-follicular pustules ranging between 2 and 3 mm in size. These pustules showed a tendency to coalesce, forming “lakes” of pus, particularly over the lower limbs and abdomen. As the lesions evolved, they dried and left behind residual erythema and desquamation, which was especially evident on the back and right arm. A Generalized Pustular Psoriasis Physician Global Assessment (GPPGA) score was calculated based on erythema, pustulation, and scaling, with each component scored 3 out of 4, giving a mean severity score of 3.3.

**Fig 3:**
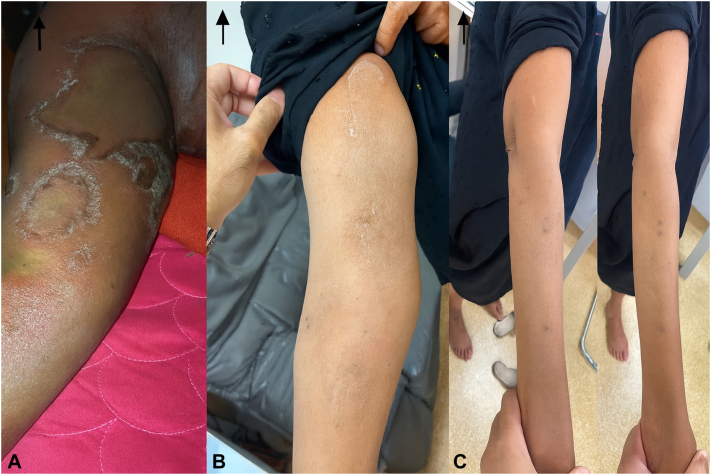
**A-C,** Pre- and post-treatment (day 10 and day 31): Right arm images.

**Fig 4:**
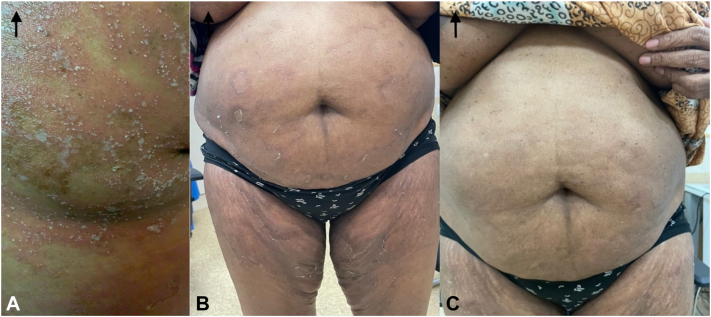
**A-C,** Pre- and post-treatment (day 10 and day 31): Abdomen images.

**Fig 5:**
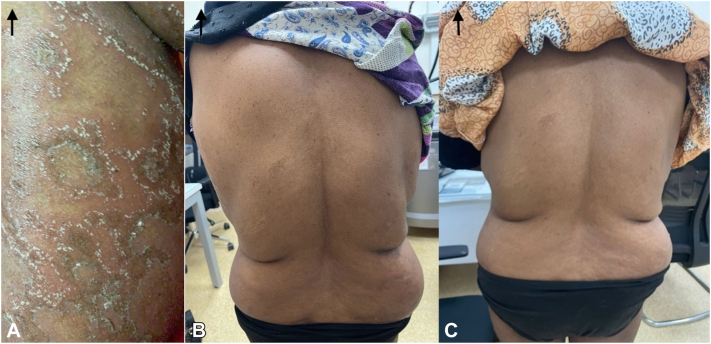
**A-C,** Pre- and post-treatment (day 10 and day 31): Back images.

**Fig 6:**
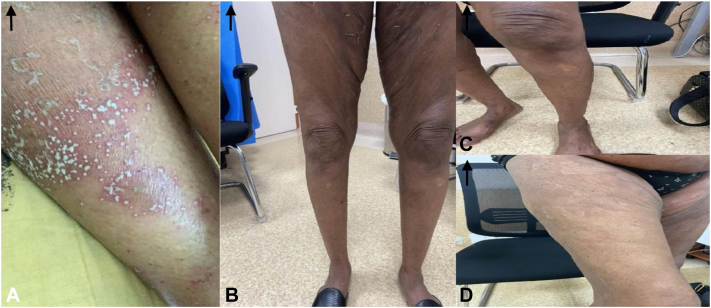
**A-D,** Pre- and post-treatment (day 10 and day 31): Lower limb images.

Ten days after receiving Spesolimab (on the 8th of December), the patient reported marked clinical improvement. She denied any recurrence of pustules, erythema, or itchiness. Furthermore, systemic symptoms such as fever and chills—commonly observed during GPP flare-ups—were absent. Physical examination corroborated her report, with no evidence of pustules or erythema and only minor residual desquamation. The GPPGA score had significantly improved to a mean of 0.33 (erythema: 0, pustules: 0, scaling: 1) (Fig 3, Fig 4, Fig 5 to 6).

At the 31-day follow-up on the 29th of December, the patient's skin was completely clear, with no residual pustules, erythema, or scaling. Systemic symptoms remained absent. A repeat GPPGA assessment showed a perfect score of 0 across all domains (mean score: 0), indicating complete clinical remission of the GPP flare-up following a single intravenous dose of Spesolimab (Fig 3, Fig 4, Fig 5 to 6). Furthermore, the patient reported sustained clearance of skin lesions and complete absence of pruritus from the time of the flare-up dose until June 2025, mirroring the results observed at the 31-day follow-up.

## Discussion

GPP is a rare and severe form of psoriasis characterized by widespread pustules and systemic symptoms. Traditional therapies, including corticosteroids, retinoids, cyclosporine, and tumor necrosis factor alpha inhibitors, often yield inconsistent results and may not be well tolerated.^5^ Increasing evidence implicates the IL-36 pathway in GPP pathogenesis, making IL-36 inhibition an attractive therapeutic target.^3^ Spesolimab, a monoclonal antibody against the IL-36 receptor, has recently demonstrated rapid efficacy in GPP flares.^4^

Two pivotal trials have shaped our understanding of spesolimab. In **Effisayil-1**, a single intravenous dose produced significant short-term improvements, with rapid pustular clearance observed within 1 week.^6^ However, durability beyond the acute flare was limited, and many patients required additional therapy. In contrast, **Effisayil-2** demonstrated that long-term disease control typically required **maintenance infusions every 4 weeks**.^10^ These findings suggest that while a single dose is highly effective for acute flares, sustained remission usually depends on repeated dosing.

Our patient’s course differs from these results. After a single 900 mg infusion of spesolimab, she achieved complete clearance within 1 month and, notably, maintained remission for over **6 months without any maintenance therapy**. This outcome highlights potential variability in patient responses. Similar long-lasting responses have occasionally been reported. Brigenti et al described a woman with refractory GPP who achieved 12 months of remission after a single dose.^7^ Morita et al and Elewski et al also observed substantial improvements in subsets of patients without early retreatment.^8^^,^^9^ These findings, together with our case, suggest that for some patients, durable disease control may be achievable with a single infusion.

The clinical implications are important. If certain patients can maintain remission without repeated dosing, this approach could reduce treatment burden, lower healthcare costs, and minimize risks associated with prolonged immunosuppression. At the same time, most trial data indicate that regular maintenance is required, and vigilance regarding safety—particularly infections—remains necessary. Larger prospective studies are needed to identify predictors of durable response and to clarify which patients may benefit from a single-dose strategy versus those requiring ongoing therapy.

## Conflicts of interest

None disclosed. The preparation, writing, and conclusions presented in this case report on the use of Spesolimab for a GPP flare were conducted independently and without external influence.

## References

[1] Rivera-Díaz R., Daudén E., Carrascosa J.M., De La Cueva P., Puig L. (2023). Generalized pustular psoriasis: a review on clinical characteristics, diagnosis, and treatment. Dermatol Ther.

[2] Choon S.E., Navarini A.A., Pinter A. (2022). Clinical course and characteristics of generalized pustular psoriasis. Am J Clin Dermatol.

[3] Sachen K.L., Greving C.N.A., Towne J.E. (2022). Role of IL-36 cytokines in psoriasis and other inflammatory skin conditions. Cytokine.

[4] Bernardo D., Thaçi D., Torres T. (2023). Spesolimab for the treatment of generalized pustular psoriasis. Drugs.

[5] Krueger J., Puig L., Thaçi D. (2022). Treatment options and goals for patients with generalized pustular psoriasis. Am J Clin Dermatol.

[6] Bachelez H., Choon S.E., Marrakchi S. (2021). Trial of spesolimab for generalized pustular psoriasis. New Engl J Med.

[7] Brigenti N., Gisondi P., Bellinato F., Girolomoni G. (2024). Generalized pustular psoriasis successfully treated with spesolimab: a case report. SAGE Open Med Case Rep.

[8] Morita A., Tsai T., Yee E.Y.W. (2022). Efficacy and safety of spesolimab in Asian patients with a generalized pustular psoriasis flare: results from the randomized, double-blind, placebo-controlled Effisayil^TM^ 1 study. J Dermatol.

[9] Elewski B.E., Lebwohl M.G., Anadkat M.J. (2023). Rapid and sustained improvements in Generalized Pustular Psoriasis Physician Global Assessment scores with spesolimab for treatment of generalized pustular psoriasis flares in the randomized, placebo-controlled Effisayil 1 study. J Am Acad Dermatol.

[10] Rega F., Trovato F., Bortone G., Pellacani G., Richetta A., Dattola A. (2024). Therapeutic potential of Spesolimab-SBZO in the management of generalized pustular psoriasis flares in adults: evidence to date. Psoriasis (Auckl).

